# Classical Versus Bayesian Error-Controlled Sampling Under Lognormal Distributions with Type II Censoring

**DOI:** 10.3390/e27050477

**Published:** 2025-04-28

**Authors:** Huasen Zhou, Wenhao Gui

**Affiliations:** School of Mathematics and Statistics, Beijing Jiaotong University, Beijing 100044, China; 22271084@bjtu.edu.cn

**Keywords:** reliability testing, Bayesian sampling, failure-censored sampling, Type II censoring, lognormal distribution

## Abstract

This paper presents a comparative study of classical and Bayesian risks in the design of optimal failure-censored sampling plans for lognormal lifetime models. The analysis focuses on how variations in prior distributions, specifically the beta distribution for defect rates, influence the producer’s and consumer’s risks, along with the optimal sample size. We explore the sensitivity of the sampling plan’s risks to changes in the prior mean and variance, offering insight into the impacts of uncertainty in prior knowledge on sampling efficiency. Classical and Bayesian approaches are evaluated, highlighting the trade-offs between minimizing sample size and controlling risks for both the producer and the consumer. The results demonstrate that Bayesian methods generally provide more robust designs under uncertain prior information, while classical methods exhibit greater sensitivity to parameter changes. A computational procedure for determining the optimal sampling plans is provided, and the outcomes are validated through simulations, showcasing the practical implications for quality control in reliability testing and industrial applications.

## 1. Introduction

Type II censoring is a commonly used censoring method, especially in reliability testing and survival analysis. It is defined as the situation where an experiment or trial is terminated after a predetermined time or a certain number of samples. In experiments with time or cost constraints, Type II censoring provides an efficient and resource-saving experimental design approach, especially in fields such as reliability testing and survival analysis. By setting an upper limit on the number of failures, it ensures that the experiment can obtain valid statistical inferences within limited resources while maintaining reliability and accuracy. Since 1950, many authors have studied this topic. Parameter estimation methods for various distributions under Type II censoring were provided by [1], providing significant support to future researchers. In recent years, several studies have explored this topic [2,3,4,5,6,7,8].

Failure-censored sampling plans aim to estimate the reliability parameters of a population by sampling product lifetimes or failure times. This problem has been extensively studied, particularly in the development of lifetime models and the optimization of sampling design methods. The earliest research on failure-censored sampling was introduced in [9], laying the foundation for subsequent advancements in this area. In recent years, further research has been conducted on sampling plans for different lifetime models. For instance, reliability design methods with double censoring, applicable to certain two-parameter distributions, were introduced in [10], extending the applicability of failure-censored sampling. Moreover, in [11], sampling schemes optimized for lifetime distributions characterized by log-location–scale parameters were explored, leading to the development of design approaches suitable for more sophisticated lifetime models. Additionally, various computational methods and optimization strategies for sampling design under the Weibull distribution were proposed in [12], particularly for designing efficient sampling plans in higher-dimensional and more complex scenarios. Furthermore, the determination of the optimal reliability acceptance sampling plan under cost constraints within the hybrid censoring framework was explored in [13], addressing the challenge of balancing reliability and cost efficiency. In [14], an acceptance sampling scheme utilizing the lifetime performance index was proposed for exponential populations with and without censoring. The method achieved similar performance compared to the approximation approach based on full-order observed exponential data. Similarly, in [15], an optimal reliability acceptance sampling plan was designed using the Type I generalized hybrid censoring scheme for non-repairable products, while a cost function approach was introduced for products with Weibull-distributed lifetimes. In addition, ref. [16] introduced two innovative variable reliability acceptance sampling plans, namely, repetitive group sampling and resubmitted sampling, both of which are suitable for reliability tests under failure-censoring. Building on this progress, a variable multiple-dependent-state sampling scheme based on the Weibull distribution with Type II right censoring was proposed in [17], incorporating the lifetime performance index. Compared to the single-variable sampling scheme, the proposed approach demonstrates higher cost efficiency and greater discriminatory power. In summary, failure-censored sampling plans require significantly fewer samples than attribute sampling plans, while also considerably reducing test time compared to complete variable sampling schemes. This makes failure-censored sampling plans highly valuable for reliability testing and quality control under resource constraints.

Traditional acceptance sampling typically assumes that the proportion of nonconforming items, denoted as *p*, is a fixed value for each production batch. However, in practical applications, the true value of *p* is usually unknown prior to inspection, and it may vary across different batches due to differences in materials, processing conditions, or environmental factors. If this uncertainty is ignored, classical methods that assume a known *p* may lead to suboptimal or misaligned sampling plans. To address this issue, Bayesian methods have been introduced. In the Bayesian framework, *p* is still assumed to be fixed for a given batch, but treated as a random variable to represent our uncertainty about its true value. A Beta distribution is commonly used as the prior for *p*, due to its flexibility on the interval [0,1] and its conjugate relationship with the binomial distribution, which simplifies posterior computation. The application of Beta priors for optimizing sampling schemes was first proposed in [18] and has since provided a foundational framework for Bayesian-based acceptance sampling.

In recent years, Bayesian sampling has continued to receive widespread attention and extensive research. For instance, in [19], the application of Bayesian sampling in acceptance sampling was significantly expanded by exploring various prior distributions for the defective rate *p*, which not only enriched the theoretical framework but also enhanced practical implementations. In [20], a systematic comparison of conventional and Bayesian sampling methods was conducted. This study provided an in-depth evaluation of both consumer and producer risks while examining the sensitivity of prior distribution parameter variations. These findings contributed to a more comprehensive understanding of Bayesian sampling performance and its practical implications. As research in this field progressed, in [21], a novel acceptance sampling plan was introduced to determine whether the received lot met the predefined acceptance criteria. This approach integrated a cost objective function that accounted for potential inspection errors. Additionally, Bayesian inference was applied to refine the probability distribution function of the nonconforming proportion, whereas a backward recursive approach was employed to evaluate terminal costs and derive optimal decisions. Expanding Bayesian sampling methodologies further, in [22], two Bayesian accelerated acceptance sampling plans were proposed for a lognormal lifetime distribution under Type I censoring. The first plan incorporated risk considerations for both producers and consumers, while the second exclusively focused on consumer risk. Moreover, a sensitivity analysis was conducted to assess the impact of prior distribution selection, thereby enhancing the robustness and applicability of these models. In [23], a Bayesian reliability sampling plan was presented for the Weibull distribution under progressively Type II censoring. The study identified the optimal strategy by analyzing sample sizes, recorded failure counts, binomial-based removal probabilities, and the minimum reliability threshold. This refinement further advanced Bayesian reliability sampling techniques, making them more adaptable to real-world reliability assessments. Most recently, in [24], a Bayesian double-group sampling plan was introduced to estimate the average number of nonconforming products. By incorporating Bayesian inference principles, this approach contributed to the growing body of research focused on improving the efficiency and accuracy of Bayesian-based acceptance sampling strategies.

This paper analyzes the average and posterior risks in designing optimal failure-censored sampling plans for lognormal lifetime models, where the defect rate *p* follows a beta distribution. In Section 2, we derive the operating characteristic function for the lognormal distribution under Type II censoring, which is used to evaluate the acceptance probability of products based on the lognormal lifetime distribution. Through large-sample approximations, we estimate the impact of censoring rate and defect probability on acceptance decisions, providing a theoretical foundation for optimizing sampling plans. In Section 3, we first introduce the producer’s risk and consumer’s risk under both classical and Bayesian sampling frameworks. Then, we present a computational procedure for determining the optimal sample size and decision threshold. Finally, we analyze the properties of prior distributions under different parameter settings and explore their influence on the optimization of sampling schemes. In Section 4, we compute the optimal sample sizes corresponding to different prior distribution parameters and conduct a sensitivity analysis on both classical and Bayesian sampling methods. Based on these results, we identify the optimal sampling scheme for specific prior distributions, balancing sample size and risk control. In Section 5, we synthesize the graphs and simulation results obtained in Section 4 to draw conclusions. We further provide recommendations for producers and consumers regarding the application of optimal sampling schemes. The findings contribute to reliability testing and quality control, aiding in the development of more robust sampling strategies.

## 2. Operating Characteristic Function

The lognormal distribution is one of the most widely used methods of survival analysis, reliability analysis, and sampling inspection. In reliability testing, suppose the lifetime *T* of a batch of electronic components follows a two-parameter lognormal distribution. The logarithm of the lifetime variable, X=log(T), follows a normal distribution with an unknown location parameter μ and scale parameter σ. In this article, we primarily study the logarithmic lifetime *X*.

The cumulative distribution function (CDF), probability density function (PDF), and survival function (SF) of the random variable *X* are given by:

$$F\left(x;\mu ,\sigma \right)=\Phi \left(\frac{x-\mu }{\sigma }\right),$$

$$f\left(x;\mu ,\sigma \right)=\varphi \left(x\right)=\frac{1}{\sqrt{2\pi }\sigma }\exp\left(-\frac{{\left(x-\mu \right)}^{2}}{2\sigma^{2}}\right),$$

$$S\left(x;\mu ,\sigma \right)=1-F\left(x;\mu ,\sigma \right)=1-\Phi \left(\frac{x-\mu }{\sigma }\right).$$

In the following, Φ(·) represents the standard normal distribution function, and φ(x) represents the probability density function.

Given a total sample size of *n*, the test is terminated upon the failure of the *m*-th component. At this point, the logarithmic lifetime $X_{\left(1\right)}\leq X_{\left(2\right)}\leq \cdots \leq X_{\left(m\right)}$ is recorded, while the remaining n−m components have not yet failed and are considered censored, with a censoring rate defined as $q=1-\frac{m}{n}$, representing the proportion of units that are censored due to not failing within the test duration. This parameter will be crucial in later derivations. Consequently, only the first *m* order statistics $X_{\left(1\right)},\ldots ,X_{\left(m\right)}$ are available, and it is known that n−m units remain beyond $X_{\left(m\right)}$.

When $X_{i}$ is independently and identically distributed as $N(\mu ,\sigma^{2})$ and only the first *m* order statistics $X_{\left(1\right)},\ldots ,X_{\left(m\right)}$ are available, their joint density function is:

$$f_{x_{1},x_{2},\ldots ,x_{m}}\left(x_{\left(1\right)},\ldots ,x_{\left(m\right)};\mu ,\sigma \right)=\frac{n!}{(n-m)!}\prod_{i=1}^{m}f_{X}\left(x_{\left(i\right)}\right){\left[S_{X}\left(x_{\left(m\right)}\right)\right]}^{n-m}.$$

Here, the faction $\frac{n!}{(n-m)!}$ arises from the joint density of order statistics and reflects the number of ways to arrange *m* failure times among *n* total units, with the remaining n−m units being censored.

The lifetime variable is assumed to follow a lognormal distribution, which is a type of log-location–scale lifetime model. Therefore, we apply a logarithmic transformation to the data and perform all subsequent analysis on the logarithmic scale, including the logarithmic lifetime *X* and the lower limit *ℓ*. Under this transformation, the lifetime variable becomes normally distributed, allowing us to formulate the model within the framework of the normal distribution. This transformation does not alter the acceptance decision criterion for the products, and it also enables us to take advantage of the well-established theoretical results for Type II censoring under the normal distribution.

For an electronic component to meet quality standards, its lifetime must exceed a given lower limit *ℓ*. If x<𝓁, the product is considered defective, commonly referred to as a “bad value” in quality control. The defect rate *p* is defined as:

Pr(X<𝓁)=p.

Since $X∼N(\mu ,\sigma^{2})$, we have $\mathrm{Pr}\left(X<𝓁\right)=\Phi \left(\frac{𝓁-\mu }{\sigma }\right)=p$; hence, solving for *ℓ* yields:

$$𝓁=\mu +\sigma \Phi^{-1}\left(p\right).$$

A batch of products is accepted if the maximum likelihood estimates (MLEs) $\hat{\mu }$ and $\hat{\sigma }$ under Type II censoring satisfy:

$$\hat{\mu }-k\hat{\sigma }>𝓁,$$

where *k* is a predetermined constant. Substituting *ℓ*, we obtain $\frac{\hat{\mu }-\mu }{\sigma }-k\frac{\hat{\sigma }}{\sigma }>\Phi^{-1}\left(p\right)$. Defining the pivotal quantities $Y_{1}=\frac{\hat{\mu }-\mu }{\sigma }$ and $Y_{2}=\frac{\hat{\sigma }}{\sigma }$, the inequality simplifies to $Y_{1}-kY_{2}>\Phi^{-1}\left(p\right)$. Thus, the operating characteristic (OC) curve is defined as:

(1) $$L\left(p\right)=\mathrm{Pr}\left(\hat{\mu }-k\hat{\sigma }>𝓁\right)=\mathrm{Pr}\left(Y_{1}-kY_{2}>\Phi^{-1}\left(p\right)\right),\;0<p<1.$$

As shown in [25], the asymptotic normal approximation of the OC function L(·) is reasonable for the lognormal case under Type II censoring:

$$\sqrt{n}{\left(\begin{array}{c} Y_{1},Y_{2}-1 \end{array}\right)}^{⊤}\overset{d}{\to }N\left(0,\Sigma \right),$$

The notation $\overset{d}{\to }N\left(0,\Sigma \right)$ indicates that, as the sample size n→∞, the random vector composed of two statistics,

$$\sqrt{n}\left(\begin{array}{c} Y_{1}\\ Y_{2}-1 \end{array}\right),$$

converges in distribution to a bivariate normal distribution with zero mean and covariance matrix Σ. Here, $Y_{1}=\left(\hat{\mu }-\mu \right)/\sigma$ represents the standardized deviation of the MLE for the location parameter, and $Y_{2}-1=\left(\hat{\sigma }-\sigma \right)/\sigma$ denotes the relative deviation of the MLE for the scale parameter.

Here, Σ is a 2×2 covariance matrix with elements $\gamma_{ij}$ (i,j=1,2) derived from the inverse of the Fisher information matrix under Type II censoring.

Let the information matrix be:

$$J=\left(\begin{array}{cc} J_{11} & J_{12}\\ J_{12} & J_{22} \end{array}\right),$$

Then:

$$\Sigma =\left(\begin{array}{cc} \gamma_{11} & \gamma_{12}\\ \gamma_{12} & \gamma_{22} \end{array}\right)=\frac{1}{J_{11}J_{22}-J_{12}^{2}}\left(\begin{array}{cc} J_{22} & -J_{12}\\ -J_{12} & J_{11} \end{array}\right).$$

Defining $Z=Y_{1}-k\left(Y_{2}-1\right)$, its asymptotic distribution is given by:

$$\sqrt{n}Z∼N\left(0,\gamma_{11}-2k\gamma_{12}+k^{2}\gamma_{22}\right),$$

Thus, the decision rule is rewritten as $Z>\Phi^{-1}\left(p\right)+k$. By the asymptotic normality,

$$\mathrm{Pr}\left(Y_{1}-kY_{2}>\Phi^{-1}\left(p\right)\right)\approx 1-\Phi \left(\frac{\sqrt{n}\left(\Phi^{-1}\left(p\right)+k\right)}{\sqrt{\gamma_{11}-2k\gamma_{12}+k^{2}\gamma_{22}}}\right).$$

Thus, the approximate expression for the OC curve under Type II censoring is:

(2) $$L\left(p;n,m,k\right)\approx 1-\Phi \left(\lambda_{p,n,m,k}\right),\;0<p<1,$$

where

$$\lambda_{p,n,m,k}=\frac{\sqrt{n}\left(\Phi^{-1}\left(p\right)+k\right)}{\sqrt{\gamma_{11}-2k\gamma_{12}+k^{2}\gamma_{22}}}.$$

For $\left(\hat{\mu },\hat{\sigma }\right)$ under Type II censoring in a normal distribution, the asymptotic variance–covariance matrix elements correspond to the negative expected values of the second-order partial derivatives of the log-likelihood function concerning the parameters. According to [1], if $x∼N(\mu ,\sigma^{2})$ and

$$\Phi_{11}\left(q,\xi \right)=1+\Omega \left(q,\xi \right)\left[Q\left(-\xi \right)+\xi \right],$$

$$\Phi_{12}\left(q,\xi \right)=\Omega \left(q,\xi \right)\left[1+\xi Q\left(-\xi \right)+\xi \right],$$

$$\Phi_{22}\left(q,\xi \right)=2+\xi \Phi_{12}\left(q,\xi \right),$$

In Type II censoring, the truncation point is denoted by *w*, and the standardized truncation point, represented as ζ, is defined by $\zeta =\frac{w-\mu }{\sigma }$. The function Q(x) is given by $Q\left(x\right)=\frac{\varphi (x)}{1-\Phi (x)}$, while Ω(h,ζ) is expressed as $\Omega \left(h,\zeta \right)=\frac{h}{1-h}Q\left(-\zeta \right)$.

We have:

$$\gamma_{11}=\mathrm{Var}\left(\hat{\mu }\right)=\frac{\sigma^{2}}{n}\mu_{11},\;\gamma_{22}=\mathrm{Var}\left(\hat{\sigma }\right)=\frac{\sigma^{2}}{n}\mu_{22},$$

$$\gamma_{12}=\gamma_{21}=\mathrm{Cov}\left(\hat{\mu },\hat{\sigma }\right)=\frac{\sigma^{2}}{n}\mu_{12}.$$

Here, $\mu_{11},\mu_{12},\mu_{22}$ are functions of $\Phi_{11},\Phi_{12},\Phi_{22}$, which are elements of the information matrix. In the case of Type II censoring with a total sample size of *n* and a censoring rate of *q*, the calculation methods for $\mu_{11},\mu_{12},\mu_{22}$ are as follows:

$$\mu_{11}=\left(\frac{n}{m}\right)\frac{\Phi_{22}}{\Phi_{11}\Phi_{22}-\Phi_{12}^{2}},$$

$$\mu_{22}=\left(\frac{n}{m}\right)\frac{\Phi_{11}}{\Phi_{11}\Phi_{22}-\Phi_{12}^{2}},$$

$$\mu_{12}=\left(\frac{n}{m}\right)\frac{\Phi_{12}}{\Phi_{11}\Phi_{22}-\Phi_{12}^{2}}.$$

The following diagram illustrates the relationship between $\Phi_{11}$, $\Phi_{12}$, $\Phi_{22}$ and the elements $\gamma_{ij}$ in the covariance matrix Σ.

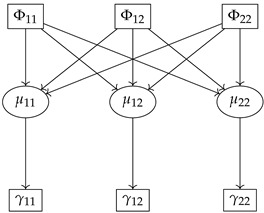



In practical applications, the probability bounds for the product failure rate *p* are typically determined according to industrial standards. These bounds define the region over which the producer’s and consumer’s risks are assessed, and they further guide the determination of the sample size *n*, the number of failures *m*, and the predetermined constant *k* for the test statistic. A more detailed mathematical formulation of this design process is provided in Section 3.

## 3. Risk Criteria and Prior Models

In reliability testing and quality control, both producers and consumers need to jointly establish quality standards based on the defect rate *p* to ensure that the production process controls the outflow of nonconforming products while not being overly stringent on conforming products. The acceptable quality level and the rejectable quality level are two key indicators that represent the highest defect rate $p_{\tau_{1}}$ acceptable to the producer and the lowest defect rate $p_{\tau_{2}}$ unacceptable to the consumer, respectively, where $p_{\tau_{2}}>p_{\tau_{1}}$.

In quality inspection, sampling tests determine whether a production batch meets the required quality standards. If a batch satisfies the acceptance criteria, it is considered accepted; otherwise, it is rejected. Thus, batch acceptance indicates a passed test, while batch rejection signifies a failed test.

Based on this, the quality inspection process involves two main risks: producer’s risk (*PR*) refers to the probability that a conforming batch ($p\leq p_{\tau_{1}}$) is rejected due to sampling error, denoted as $\tau_{1}$; consumer’s risk (*CR*) refers to the probability that a nonconforming batch ($p\geq p_{\tau_{2}}$) is accepted due to sampling error, indicated as $\tau_{2}$.

The goal of the sampling plan is to determine the optimal scheme (n,m,k) (i.e., minimizing sample size) such that:

(3) $$PR\left(n,m,k\right)\leq \tau_{1},\;CR\left(n,m,k\right)\leq \tau_{2}.$$

This ensures that both producer and consumer risks are controlled, balancing the acceptance rate of conforming products and the rejection rate of nonconforming products while maintaining a reasonable sample size and testing cost. A censoring mechanism is introduced to reduce testing time or cost, and prior distributions are further incorporated to optimize decision-making. Here, $h_{1}\left(\cdot \right)$ and $h_{2}\left(\cdot \right)$ represent the prior probability density functions of *p* for the producer and consumer, respectively, with the corresponding cumulative distribution functions $H_{1}\left(\cdot \right)$ and $H_{2}\left(\cdot \right)$. Bayesian methods can further optimize the sampling strategy, making decisions statistically more rational.

### 3.1. Classical and Bayesian Risks

Different risk criteria guide the selection of the optimal sampling plan (n,m,k). Given that the probability of batch acceptance is expressed as Pr(“batchisaccepted”)=L(p;n,m,k), the classical average risks are defined as follows:

- Average Producer’s Risk (*APR*)
  $$\mathrm{Pr}\left(“\mathrm{batch}\;\mathrm{is}\;\mathrm{rejected}”|p\leq p_{\tau_{1}}\right)=E^{h_{1}}\left[1-L\left(p\right)|p\leq p_{\tau_{1}}\right]$$
  (4) $$=1-\frac{1}{H_{1}\left(p_{\tau_{1}}\right)}\int_{0}^{p_{\tau_{1}}}L\left(p\right)h_{1}\left(p\right)dp,$$
- Average Consumer’s Risk (*ACR*)
  $$\mathrm{Pr}\left(“\mathrm{batch}\;\mathrm{is}\;\mathrm{accepted}”|p\geq p_{\tau_{2}}\right)=E^{h_{2}}\left[L\left(p\right)|p\geq p_{\tau_{2}}\right]$$
  (5) $$=\frac{1}{1-H_{2}\left(p_{\tau_{2}}\right)}\int_{p_{\tau_{2}}}^{1}L\left(p\right)h_{2}\left(p\right)dp.$$

Additionally, Bayesian or posterior risks are defined as follows:

- Bayesian Producer’s Risk (*BPR*)
  (6) $$\mathrm{Pr}\left(p\leq p_{\tau_{1}}|“\mathrm{batch}\;\mathrm{is}\;\mathrm{rejected}”\right)=\frac{\int_{0}^{p_{\tau_{1}}}\left(1-L\left(p\right)\right)h_{1}\left(p\right)dp}{\int_{0}^{1}\left(1-L\left(p\right)\right)h_{1}\left(p\right)dp},$$
- Bayesian Consumer’s Risk (*BCR*)
  (7) $$\mathrm{Pr}\left(p\geq p_{\tau_{2}}|“\mathrm{batch}\;\mathrm{is}\;\mathrm{accepted}”\right)=\frac{\int_{p_{\tau_{2}}}^{1}L\left(p\right)h_{2}\left(p\right)dp}{\int_{0}^{1}L\left(p\right)h_{2}\left(p\right)dp}.$$

### 3.2. A Computational Procedure

To obtain the optimal sampling plan $\left(n,m,k^{★}\right)$ that satisfies the condition in Equation (3), we solve it using Algorithm 1. In this study, we employed the particle swarm optimization algorithm to estimate the model parameters. In the initial stage, we adopted a relatively broad search range to avoid premature convergence to local optima. Based on preliminary results, the parameter bounds were then refined. The final search intervals were set as:

n∈[2.0001,50.0],k∈[−10,10].

The remaining particle swarm optimization parameters were configured as follows: swarm size of 500, maximum number of iterations set to 100, inertia weight w=0.5, and acceleration coefficients $c_{1}=c_{2}=0.7$. A boundary-clipping mechanism was applied at each iteration to ensure that all particles remained within the feasible search space, thereby enhancing the stability and convergence of the algorithm.

After obtaining the optimal solution $\left(n_{0},k_{0}\right)$ from the equation, we take into account that the sample size *n* must be a positive integer. Therefore, we round $n_{0}$ up to the nearest integer to obtain the actual sample size *n*. Next, we substitute *n* and $k_{0}$ into Equation (3)–(7) for verification: if the condition in Equation (3) is satisfied, the value of *m* is set to its floor; otherwise, it is set to its ceiling.

Finally, the determined value of *n* is substituted into the two equations from Step 2 to compute the corresponding values $k_{\tau_{1}}$ and $k_{\tau_{2}}$, and the final value of $k^{★}$ is taken as the average of these two. A detailed description of the procedure is provided in the algorithm below.
**Algorithm 1:** Computational Procedure for Optimal Sampling Plan
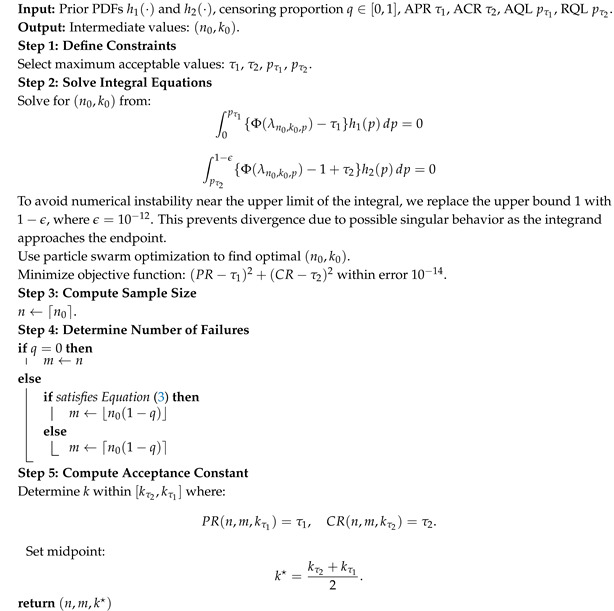


### 3.3. Prior Distribution of Defect Rate

In industrial production, the defect rate *p* of batches of electronic components is not fixed but varies randomly due to factors such as materials, processes, and environmental conditions. The overall defect rate *p* is generally unobservable, but it can be estimated based on historical data or empirical prior information. Suppose the prior probability density function of *p* follows a beta distribution:

p∼Beta(a,b),

With the probability density function given by:

(8) $$h\left(p\right)=\frac{p^{a-1}{\left(1-p\right)}^{b-1}}{B(a,b)},\;0\leq p\leq 1.$$

where B(a,b) is the beta function. When the producer’s and consumer’s risk assessments adopt the same prior information, i.e., $h_{1}\left(p\right)=h_{2}\left(p\right)$, it indicates that both parties rely on the same historical data and quality control standards. This assumption enables producers and consumers to optimize the sampling plan within a common informational framework. In the Bayesian approach, the prior mean and prior variance of the defect rate are expressed as:

$$\mu_{p}=\frac{a}{a+b},\;\sigma_{p}^{2}=\frac{ab}{{\left(a+b\right)}^{2}\left(a+b+1\right)}.$$

**Figure 1:**
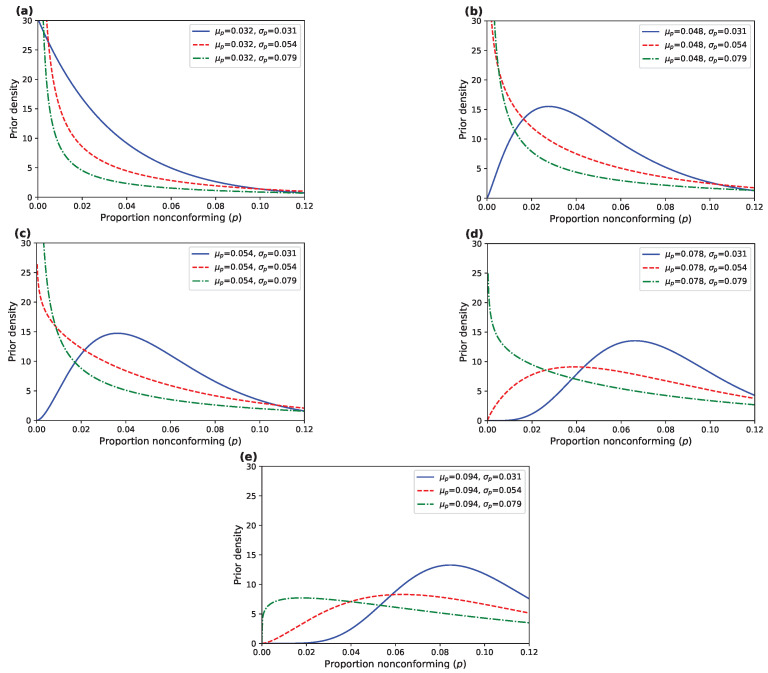
Beta(a,b) prior distributions for *p* at given $\mu_{p}$ and $\sigma_{p}$. Subfigures illustrate the influence of different prior means and variances: (**a**) Beta(a,b) prior distributions for the defect rate *p* under $\mu_{p}=0.032$ with varying prior variances; (**b**) Beta(a,b) prior distributions for the defect rate *p* under figure $\mu_{p}=0.048$ with varying prior variances; (**c**) Beta(a,b) prior distributions for the defect rate *p* under $\mu_{p}=0.054$ with varying prior variances; (**d**) Beta(a,b) prior distributions for the defect rate *p* under $\mu_{p}=0.078$ with varying prior variances; (**e**) Beta(a,b) prior distributions for the defect rate *p* under $\mu_{p}=0.094$ with varying prior variances.

Here, $\mu_{p}$ represents the prior mean of the defect rate, influencing the overall judgment of product quality by both producers and consumers, while $\sigma_{p}^{2}$ quantifies uncertainty. As *a* and *b* increase, the weight of prior information strengthens, leading to a more conservative risk assessment. Figure 1 displays several beta densities for selected values of $\mu_{p}$ and $\sigma_{p}$.

## 4. Sensitivity of Optimal Designs

In this section, we define the optimal sampling design as a triplet $\left(n,m,k^{★}\right)$ that minimizes the sample size *n* while satisfying the producer’s risk constraint $\mathrm{PR}\left(n,m,k\right)\leq \tau_{1}$ and the consumer’s risk constraint $\mathrm{CR}\left(n,m,k\right)\leq \tau_{2}$. The computational procedure proposed in Section 3 is designed to search for such optimal designs under various assumptions on the prior distributions. We primarily investigate the impact of variations in the prior beta distribution’s mean $\mu_{p}$ and standard deviation $\sigma_{p}$ on producer and consumer risks and conduct a sensitivity analysis of $\mu_{p}$ and $\sigma_{p}$.

In the following sampling schemes, we consider a lognormal sampling process with a censoring rate of 50%, where $\tau_{1}=0.05$ and $\tau_{2}=0.10$. Table 1 also includes simulated producer and consumer risks corresponding to the optimal design solutions. In electronic component manufacturing and quality control, the defect rate *p* of product batches is not fixed but fluctuates due to factors such as raw materials, process stability, and environmental conditions. Consequently, relying on a single-point estimate may lead to producer risk and consumer risk exceeding acceptable limits, affecting the effectiveness of quality management.

To better characterize the uncertainty in *p*, we introduce the beta distribution as a prior distribution. The prior mean $\mu_{p}$ represents the average defect rate in the production process, while the prior variance $\sigma_{p}$ quantifies the extent of fluctuations in the defect rate. A smaller $\sigma_{p}$ indicates a more stable production process, whereas a larger $\sigma_{p}$ suggests greater quality variations, necessitating a stricter inspection strategy.

Based on the [26] quality standard, we select $p_{\tau_{1}}=0.0319$ and $p_{\tau_{2}}=0.0942$ as quality control parameters and analyze the impact of different $\mu_{p}$ and $\sigma_{p}$ values on producer and consumer risks. This ensures that even with imperfect prior information, the sampling plan can be optimized to balance the interests of both producers and consumers while enhancing the robustness of quality management.

The selected values of $\mu_{p}$ lie within the interval $\left(p_{\tau_{1}},p_{\tau_{2}}\right)$, while $\sigma_{p}$ is proportional to the length of this interval. The prior mean values of *p*, set at 0.032, 0.063, and 0.094, represent “low”, “medium” and “high” levels, respectively. The additional values of 0.048 and 0.078 allow us to better understand the trend of changes in $\mu_{p}$, enabling a more detailed sensitivity analysis in subsequent steps. Similarly, $\sigma_{p}$ is chosen as 0.047, 0.054, and 0.062, representing “low”, “medium” and “high” levels’ values, respectively.

For the Bayesian risk scheme, when $\mu_{p}=0.032$, this value is too close to 0.0319, making the requirements overly stringent. The resulting sample size n<5 is unrealistically small and lacks practical significance in real-world sampling, so specific results are not provided in the table. Observing the overall trend, we can see that, under the same prior distribution, the average risk method requires a larger sample size compared to the Bayesian risk approach. This outcome aligns with our expectations.

As discussed earlier, the optimal sampling plan proposed in this study is based on the large-sample theory. To evaluate the effectiveness of these asymptotic solutions, we perform a Monte Carlo simulation study. A detailed description of the simulation methodology can be found in [11]. The general procedure is as follows: First, we generate 5000 values of *p* following the current prior beta distribution. Then, we generate 5000 sets of samples from a lognormal distribution, each containing *n* observations. Subsequently, risk simulations are performed under a censoring rate of 50%. It is evident that under the lognormal distribution, the simulated average producer risk and consumer risk closely approximate the expected risks, $\tau_{1}=0.05$ and $\tau_{2}=0.10$, respectively.

Using the results calculated in Table 1, we plot the OC curves for the lognormal distribution under classical sampling and Bayesian sampling, with a censoring rate of 50%, and a prior distribution with mean $\mu_{p}=0.063$ and standard deviation $\sigma_{p}=0.054$.

**Figure 2:**
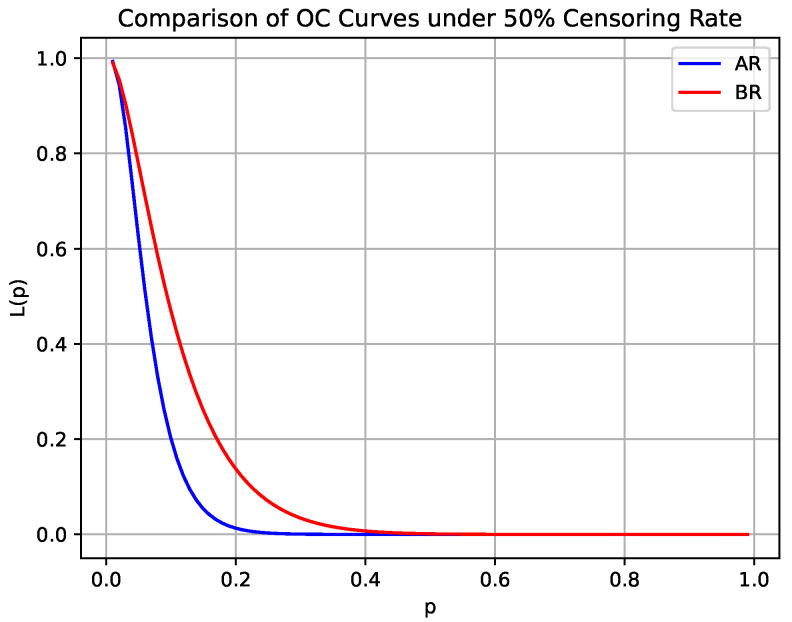
OC curves for AR design and BR design under 50% censoring rate with $\mu_{p}=0.063$ and $\sigma_{p}=0.054$.

As shown in Figure 2, the AR design exhibits a curve that rapidly approaches 0, demonstrating high sensitivity to variations in quality levels, particularly near the acceptance threshold, where the acceptance probability declines sharply. This behavior aligns with the characteristics of classical sampling, which relies on a fixed standard without incorporating data or prior distributions, thus responding quickly to quality fluctuations. In contrast, the BR design exhibits a curve that gradually approaches 0, with a higher initial acceptance probability and greater tolerance for uncertainty. By incorporating prior information and updating iteratively, the Bayesian method enhances tolerance for low-quality batches, resulting in a smoother OC curve.

### 4.1. Influence of Prior Moments on Risks

#### 4.1.1. Average Risk Design and Bayesian Risk Design

**Figure 3:**
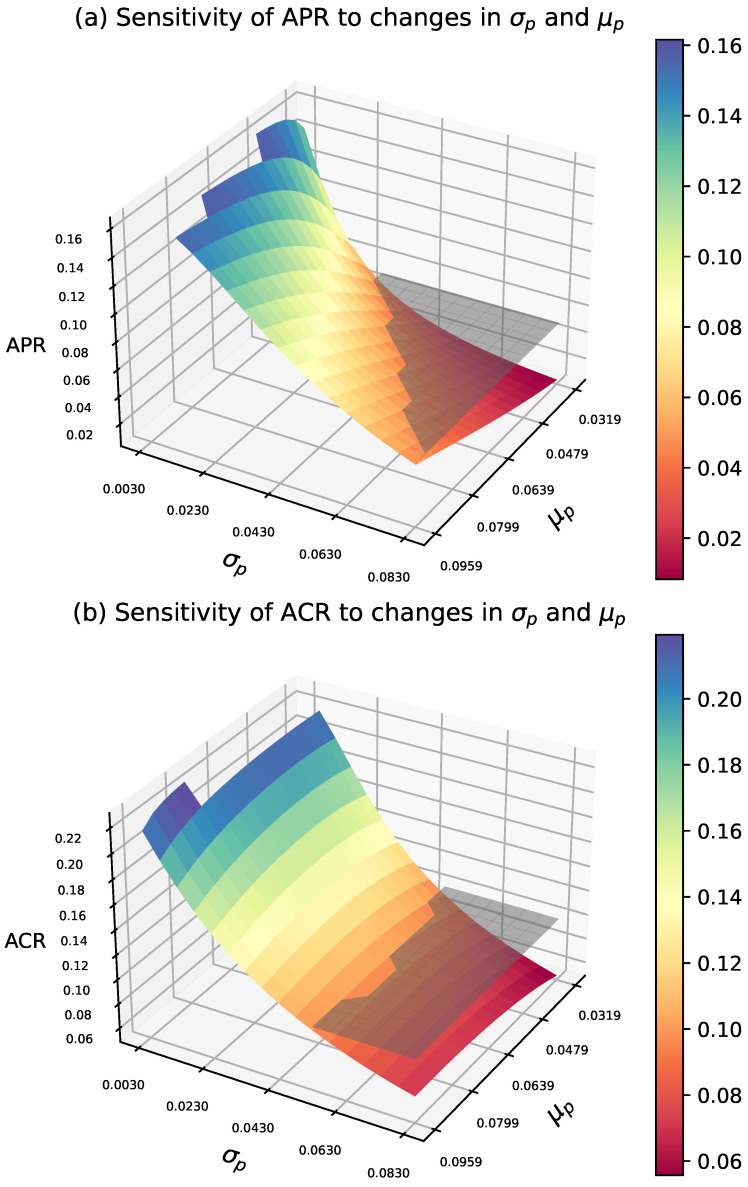
Sensitivity of average risks to $\mu_{p}$ and $\sigma_{p}$ variations in AR design, with $\tau_{1}=0.05$, $\tau_{2}=0.10$, $p_{\tau_{1}}=0.0319$, $p_{\tau_{2}}=0.0942$, $\mu_{p}=0.063$, and $\sigma_{p}=0.054$.

**Figure 4:**
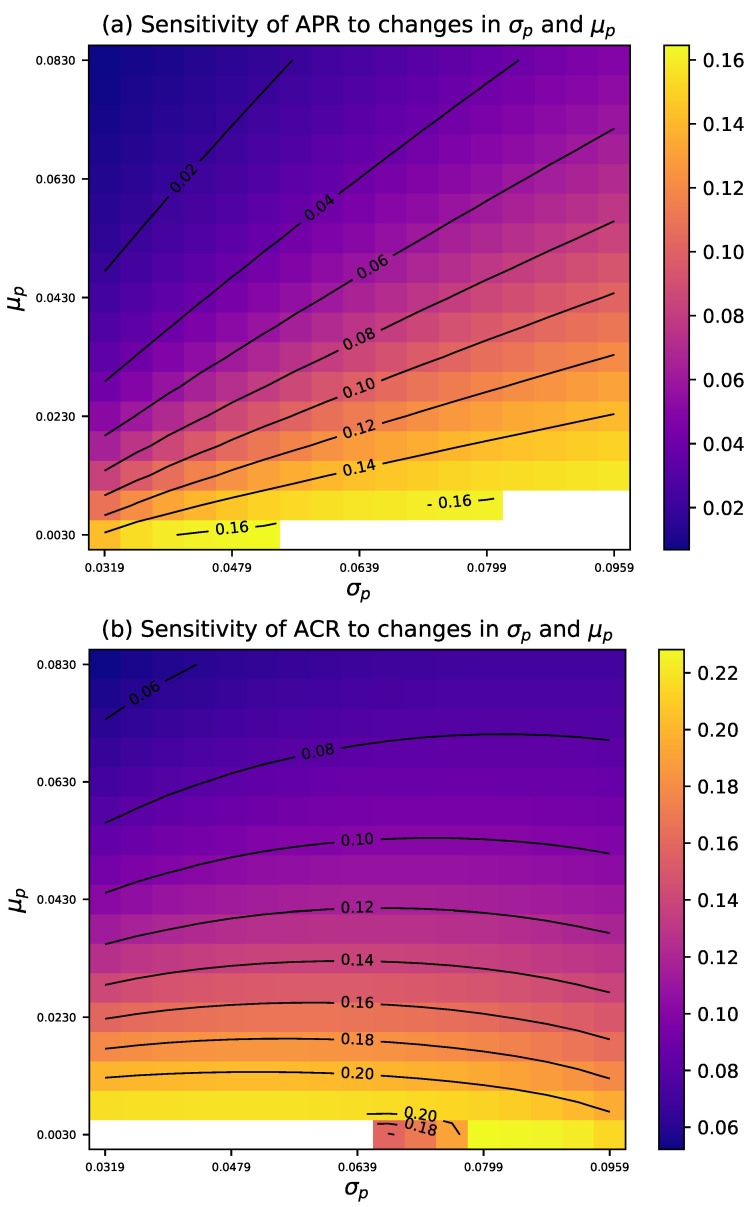
Sensitivity of average risks to $\mu_{p}$ and $\sigma_{p}$ variations in AR design, with $\tau_{1}=0.05$, $\tau_{2}=0.10$, $p_{\tau_{1}}=0.0319$, $p_{\tau_{2}}=0.0942$, $\mu_{p}=0.063$, and $\sigma_{p}=0.054$.

This study considers the optimal average risk sampling plan under given specifications, with prior $\mu_{p}=0.063$ and $\sigma_{p}=0.054$. By modifying the values of $\mu_{p}$ and $\sigma_{p}$, we illustrate their impact on design risks. To this end, Figure 3 presents the sensitivity of the average risk when $\mu_{p}$ and $\sigma_{p}$ vary simultaneously, while Figure 4 provides the corresponding contour plots.

Clearly, the producer’s risk is highly sensitive to changes in both $\sigma_{p}$ and $\mu_{p}$. In the lower range of $\sigma_{p}$, the APR initially exceeds the threshold $\tau_{1}$ and gradually decreases towards $\tau_{1}$ as $\sigma_{p}$ increases. When $\mu_{p}$ is high, the reduction in APR is less pronounced, while at lower $\mu_{p}$ values, the change is more significant. Furthermore, in the medium to high range of $\sigma_{p}$ and in lower values of $\mu_{p}$, APR decreases significantly. However, when $\sigma_{p}$ is high and $\mu_{p}$ is low, the decrease in APR slows down.

The consumer risk is similarly sensitive to variations in $\sigma_{p}$ and $\mu_{p}$. In the lower range of $\sigma_{p}$, ACR remains at a relatively high level and decreases as $\sigma_{p}$ increases. When $\mu_{p}$ is higher, the reduction in ACR is more gradual; however, in the lower $\mu_{p}$ range, the decline in ACR is more pronounced. From a three-dimensional perspective, ACR exhibits a clear downward trend as $\sigma_{p}$ increases, particularly in the low to moderate range $\mu_{p}$. In the high $\sigma_{p}$ and $\mu_{p}$ regions, changes in ACR tend to stabilize. When $\sigma_{p}$ is high, the rate of ACR reduction slows down.

**Figure 5:**
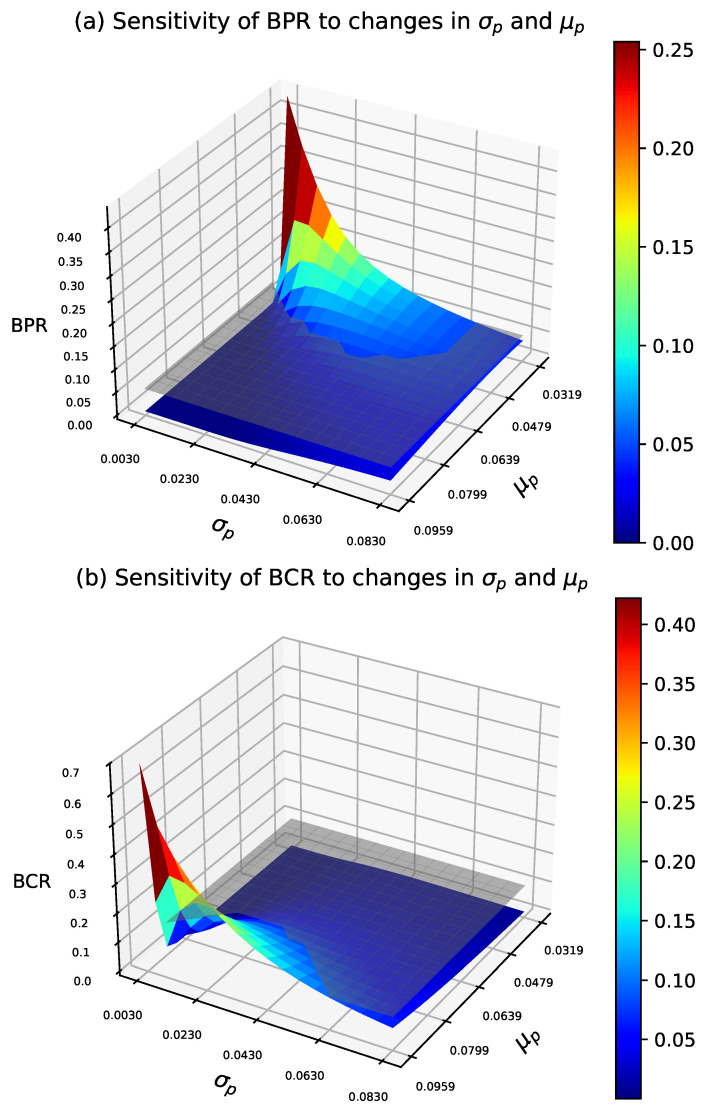
Sensitivity of Bayesian risks to $\mu_{p}$ and $\sigma_{p}$ variations in BR design, with $\tau_{1}=0.05$, $\tau_{2}=0.10$, $p_{\tau_{1}}=0.0319$, $p_{\tau_{2}}=0.0942$, $\mu_{p}=0.063$, and $\sigma_{p}=0.054$.

**Figure 6:**
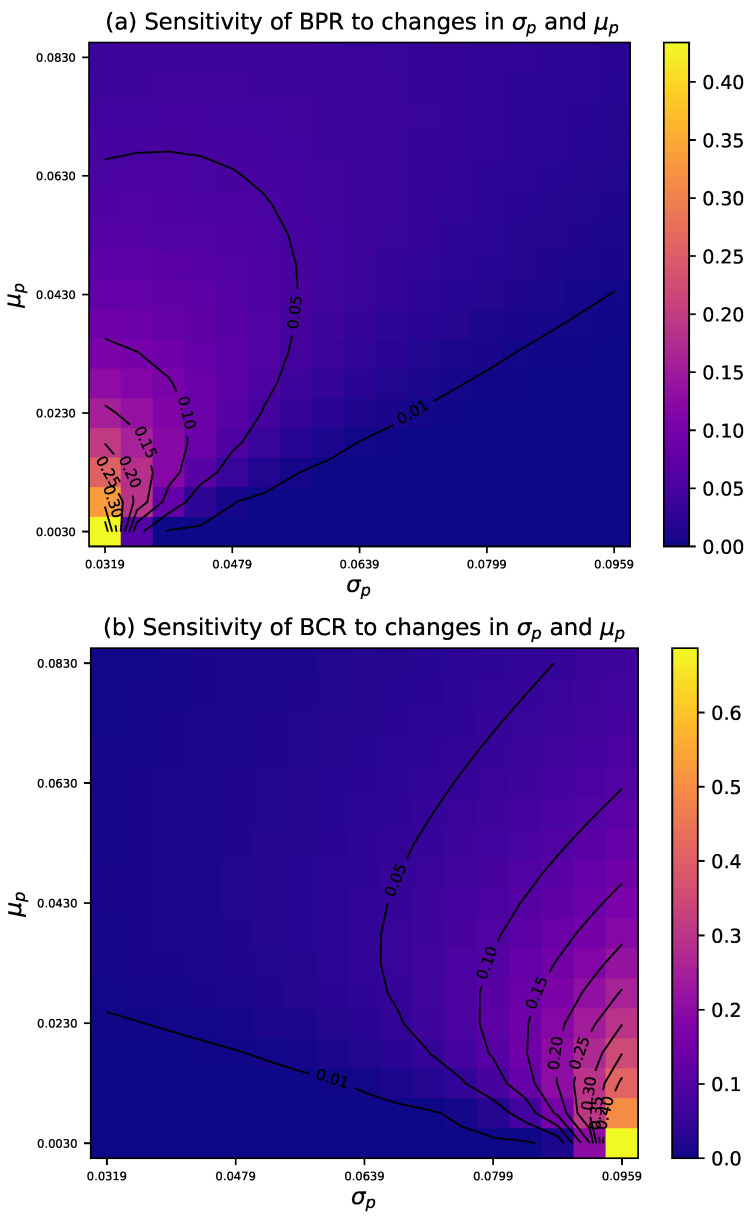
Sensitivity of Bayesian risks to $\mu_{p}$ and $\sigma_{p}$ variations in BR design, with $\tau_{1}=0.05$, $\tau_{2}=0.10$, $p_{\tau_{1}}=0.0319$, $p_{\tau_{2}}=0.0942$, $\mu_{p}=0.063$, and $\sigma_{p}=0.054$.

We analyze the Bayesian risk sampling plan under the same prior as in the AR design. Figure 5 shows how prior-moment variations affect Bayesian producer and consumer risks under the optimal design. These risks are evaluated using Equations (6) and (7) and compared with the reference planes $BPR=\tau_{1}$ and $BCR=\tau_{2}$. The contour plots are in Figure 6.

In general, for small $\sigma_{p}$, as $\mu_{p}$ increases from low to moderate values, BPR exhibits a significant decline, while BCR grows more gradually. At higher $\mu_{p}$ values, the decrease in BPR becomes more gradual, while BCR increases at a faster rate. For moderate to high values of $\sigma_{p}$, changes in BPR tend to stabilize, while BCR increases significantly, indicating a lower sensitivity of both risks to variations in $\sigma_{p}$.

#### 4.1.2. An Illustrative Example

In the following, we present a comparative analysis of the risk variations in the optimal AR and BR sampling plans when the prior mean $\mu_{p}$ and standard deviation $\sigma_{p}$ are modified. The analysis is conducted under a censoring rate of 50%, with a moderate prior mean of $\mu_{p}=0.063$ and a standard deviation of $\sigma_{p}=0.047$.

The optimal AR design is given by:

$$\left(n,m,k^{★}\right)=\left(26,13,1.54779\right),$$

and the optimal BR design is given by:

$$\left(n,m,k^{★}\right)=\left(11,5,1.31573\right).$$

Table 2 presents the impact of varying $\mu_{p}$ while keeping $\sigma_{p}=0.054$ fixed. As $\mu_{p}$ increases from 0.032 to 0.094, the producer and consumer risks exhibit different trends under the optimal AR and BR designs. The producer risk increases as $\mu_{p}$ grows, particularly in the AR design, where it rises significantly from 0.0162 to 0.0816, corresponding to an increase of approximately 49%. Similarly, the consumer risk follows an increasing trend but with a smaller growth, rising from 0.0829 to 0.0965, a relative change of 16.8%.

Under the Bayesian design, the producer risk decreases as $\mu_{p}$ increases, dropping from 0.0802 to 0.0816, showing a 49% reduction. In contrast, the Bayesian consumer risk exhibits a more substantial increase, with BCR rising from 0.0334 to 0.2479, representing a 146.8% increment. Overall, the producer risk is more sensitive to variations in $\mu_{p}$, while the consumer risk remains relatively stable. Compared to the classical design, the Bayesian design generally results in lower risks and demonstrates a higher sensitivity to risk changes as $\mu_{p}$ increases.

Table 3 presents the impact of varying $\sigma_{p}$ while keeping $\mu_{p}=0.063$ fixed. As $\sigma_{p}$ increases, the producer’s risk decreases significantly. Notably, when $\sigma_{p}$ increases from 0.036 to 0.068, the APR reduction reaches −28.4%. The consumer’s risk also decreases with increasing $\sigma_{p}$, but the magnitude of change is smaller. For example, when $\sigma_{p}$ increases to 0.068, the ACR decreases by 17.6%, indicating that increasing prior variance has some effect on consumer risk, but compared to the producer’s risk, the influence remains relatively stable.

For the Bayesian producer’s risk, the risk gradually decreases as $\sigma_{p}$ increases, but the reduction is less pronounced compared to the classical producer’s risk. The Bayesian consumer’s risk remains nearly unchanged with increasing $\sigma_{p}$, especially at higher values of $\sigma_{p}$. Even when $\sigma_{p}$ reaches 0.068, the BCR only changes by −10.2%, indicating that the Bayesian consumer’s risk is relatively insensitive to variations in prior variance. Particularly for larger values of $\sigma_{p}$, the Bayesian model’s impact on risk stabilizes.

To eliminate the influence of the censoring rate on sampling risks, we conducted additional experiments at a censoring rate of 10% and a censoring rate of 90%. Similarly, for a prior mean and standard deviation of moderate values ($\mu_{p}=0.063$, $\sigma_{p}=0.047$), we recalculated the optimal sampling plans under these two censoring rates. Theoretical computations were performed to compare the changes in risks for the AR and BR optimal sampling plans as the prior mean $\mu_{p}$ and standard deviation $\sigma_{p}$ varied.

Table 4 and Table 5 present the risk variations for the optimal AR and BR sampling plans under a censoring rate of 10%, given a prior mean and standard deviation of moderate values ($\mu_{p}=0.063$, $\sigma_{p}=0.047$). The comparison illustrates how changes in the prior mean $\mu_{p}$ and standard deviation $\sigma_{p}$ influence the risks.

The optimal AR design is given by:

$$\left(n,m,k^{★}\right)=\left(22,11,1.54781\right),$$

and the optimal BR design is given by:

$$\left(n,m,k^{★}\right)=\left(8,4,1.28515\right).$$

Table 6 and Table 7 present the risk variations for the optimal AR and BR sampling plans under a censoring rate of 90%, given a prior mean and standard deviation of moderate values ($\mu_{p}=0.063$, $\sigma_{p}=0.047$). The comparison illustrates how changes in the prior mean $\mu_{p}$ and standard deviation $\sigma_{p}$ influence the risks.

The optimal AR design is given by:

$$\left(n,m,k^{★}\right)=\left(29,15,1.54799\right),$$

and the optimal BR design is given by:

$$\left(n,m,k^{★}\right)=\left(14,7,1.31574\right).$$

By observing the risk variations in the optimal sampling plans under low, medium, and high censoring levels as the prior mean $\mu_{p}$ and standard deviation $\sigma_{p}$ change, we can conclude that the AR design exhibits higher sensitivity to parameter variations. In particular, when $\mu_{p}$ is relatively low or $\sigma_{p}$ is high, the changes in risk become more pronounced. This makes the AR design more suitable for scenarios where fine-tuned optimization is required based on subtle parameter variations.

On the other hand, the BR design demonstrates lower sensitivity, with relatively minor changes in risk values. The BR design remains more robust to parameter variations, maintaining stable risk levels. Therefore, it is better suited for environments where greater parameter variability is expected and system stability is a key requirement.

### 4.2. Optimal Sample Sizes

**Figure 7:**
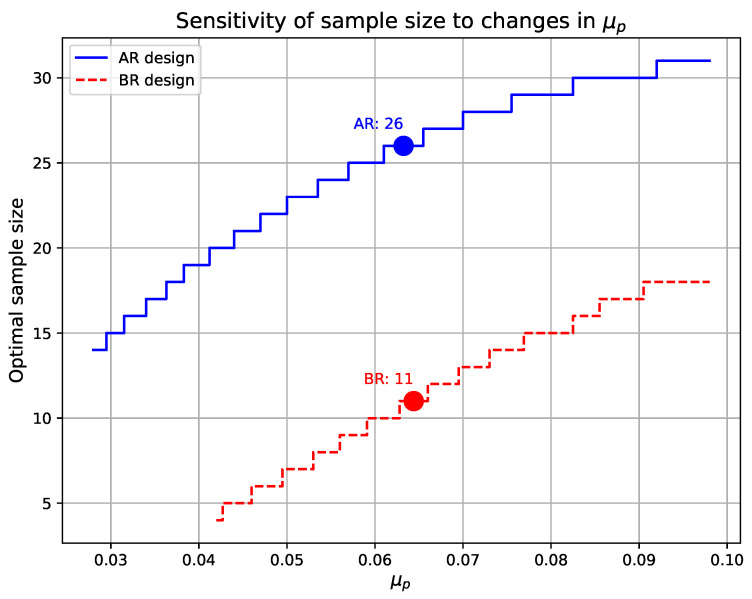
Sensitivity of optimal sample size to $\mu_{p}$ variations, with $\tau_{1}=0.05$, $\tau_{2}=0.10$, $p_{\tau_{1}}=0.0319$, $p_{\tau_{2}}=0.0942$, and $\sigma_{p}=0.054$.

In the following, we separately present the effects of changes in $\mu_{P}$ and $\sigma_{P}$ on the optimal sample size. Figure 7 illustrates the sensitivity of the optimal sample size to changes in $\mu_{P}$. When $\tau_{1}=0.05$, $\tau_{2}=0.10$, $p_{\tau_{1}}=0.0319$, $p_{\tau_{2}}=0.0942$, and $\sigma_{P}=0.054$, the figure depicts the trend of the optimal sample size for the AR and BR designs as $\mu_{P}$ varies. From the figure, it can be observed that the optimal sample size for both the AR and BR designs increases as $\mu_{P}$ increases. In contrast, the increase in sample size for the BR design is smaller, indicating that it is relatively less sensitive to changes in $\mu_{P}$.

**Figure 8:**
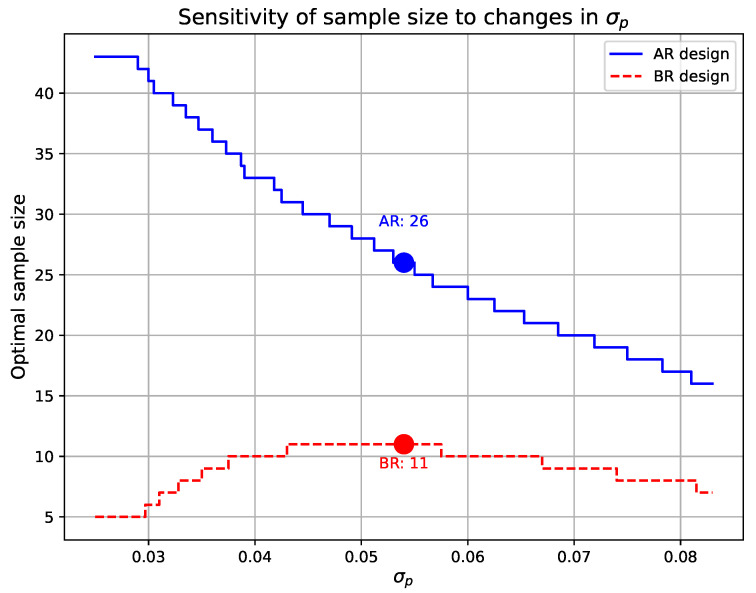
Sensitivity of optimal sample size to $\sigma_{p}$ variations, with $\tau_{1}=0.05$, $\tau_{2}=0.10$, $p_{\tau_{1}}=0.0319$, $p_{\tau_{2}}=0.0942$, and $\mu_{p}=0.063$.

Figure 8 illustrates the sensitivity of the optimal sample size to changes in $\sigma_{P}$. When $\tau_{1}=0.05$, $\tau_{2}=0.10$, $p_{\tau_{1}}=0.0319$, $p_{\tau_{2}}=0.0942$, and $\mu_{P}=0.063$, the figure shows how the AR and BR designs respond to variations in $\sigma_{P}$. From the figure, it can be observed that as $\sigma_{P}$ increases, the optimal sample size for the AR design decreases significantly in a stepwise manner, indicating that the AR design is highly sensitive to changes in the prior variance. In contrast, the BR design exhibits only minor fluctuations in sample size, demonstrating a relatively stable trend. This suggests that the BR design is more robust to variations in $\sigma_{P}$.

Additionally, we have highlighted in Figure 7 and Figure 8 the optimal sample sizes for the AR and BR designs when the prior parameters are set to $\mu_{P}=0.063$ and $\sigma_{P}=0.054$. Under these conditions, the optimal sample sizes are n=26 for the AR design and n=11 for the BR design.

## 5. Concluding Remarks

Based on the experimental data analysis, we conclude that Bayesian risk is more sensitive to $\mu_{p}$ variations than to $\sigma_{p}$ changes. When $\mu_{p}$ varies, the producer’s risk in the AR design is more affected than the consumer’s risk, whereas in the BR design, the consumer’s risk dominates. In contrast, when $\sigma_{p}$ changes, the producer’s risk fluctuates more than consumer’s risk. Additionally, the optimal sample size remains stable under minor to moderate prior moment variations. When the absolute change in $\mu_{p}$ and $\sigma_{p}$ is below 10%, sample size variations in AR and BR designs stay within 1–2 units. However, with larger $\sigma_{p}$ variations, the AR design’s sample size fluctuates significantly.

In practical production, selecting an appropriate batch sampling inspection plan requires evaluating the stability of prior parameters to determine the suitability of the AR and BR designs. When $\sigma_{P}$ is known but $\mu_{P}$ has significant uncertainty, both the producer’s and consumer’s risks are highly sensitive to this uncertainty, especially in the BR design, where the consumer’s risk fluctuates significantly. Therefore, the AR design is recommended in such cases, as it is less sensitive to changes in $\mu_{P}$ and is more suitable when $\mu_{P}$ estimation is unstable. Conversely, if $\mu_{P}$ is accurately estimated but $\sigma_{P}$ is highly variable, the producer’s risk is more affected, particularly in the AR design, where sample size variations become substantial. In this scenario, the BR design is recommended, as it offers greater stability in sample size and better controls consumer risk.

When variations in $\mu_{P}$ and $\sigma_{P}$ are small, the optimal sample size *n* should be employed for sampling, as fluctuations in sample size remain within 1–2 units, minimizing the impact on production costs. This approach is particularly suitable for stable production environments with consistent raw material quality and low equipment errors. However, when $\mu_{P}$ or $\sigma_{P}$ undergoes significant variations, especially when $\sigma_{P}$ decreases in the AR design, the optimal sample size may increase sharply. To maintain an acceptable producer’s risk, it is advisable to increase the sample size accordingly and dynamically adjust the sampling strategy. Optimizing the plan over successive batches helps mitigate quality control deviations caused by uncertainty in prior information.

Overall, in practical production, if $\sigma_{P}$ is stable but $\mu_{P}$ fluctuates, the AR design is preferable to reduce risks arising from prior mean uncertainty, making it suitable for high-risk products such as medical devices and aerospace components. Conversely, if $\mu_{P}$ is stable but $\sigma_{P}$ varies, the BR design is recommended to enhance sample size robustness and reduce sensitivity to variance deviations, making it more appropriate for products with high production consistency, such as consumer goods and electronic components.

## Figures and Tables

**Table 1:** Sample sizes and sampling risks for optimal AR and BR designs under 50% failure-censored lognormal distribution at given $\mu_{p}$ and $\sigma_{p}$, with $\tau_{1}=0.05$, $\tau_{2}=0.10$, $p_{\tau_{1}}=0.0319$, and $p_{\tau_{2}}=0.0942$.

$\mu_{p}$	$\sigma_{p}$	AR Design			BR Design		
		n	${\mathrm{APR}}_{\mathrm{sim}}$	${\mathrm{ACR}}_{\mathrm{sim}}$	n	${\mathrm{BPR}}_{\mathrm{sim}}$	${\mathrm{BCR}}_{\mathrm{sim}}$
0.032	0.047	18	0.051	0.099	–	–	–
	0.054	16	0.049	0.100	–	–	–
	0.062	13	0.048	0.103	–	–	–
0.048	0.047	25	0.051	0.096	6	0.048	0.103
	0.054	22	0.051	0.104	6	0.049	0.098
	0.062	19	0.049	0.101	6	0.051	0.108
0.063	0.047	29	0.050	0.093	11	0.054	0.099
	0.054	26	0.049	0.101	11	0.050	0.097
	0.062	23	0.051	0.972	10	0.051	0.094
0.078	0.047	32	0.052	0.098	15	0.047	0.102
	0.054	29	0.050	0.106	15	0.050	0.099
	0.062	25	0.053	0.097	14	0.049	0.098
0.094	0.047	34	0.047	0.099	20	0.047	0.097
	0.054	31	0.051	0.098	18	0.049	0.097
	0.062	27	0.054	0.102	17	0.049	0.098

**Table 2:** Effect of $\mu_{p}$ on producer and consumer risks in AR and BR designs, with $\tau_{1}=0.05$, $\tau_{2}=0.10$, $p_{\tau_{1}}=0.0319$, $p_{\tau_{2}}=0.0942$, $\mu_{p}=0.063$, and $\sigma_{p}=0.054$ under 50% censoring.

$\mu_{p}$	AR Design		BR Design	
	APR	ACR	BPR	BCR
0.032 (−49%)	0.0162 (−68.0%)	0.0829 (−15.7%)	0.0802 (57.3%)	0.0334 (−66.8%)
0.038 (−40%)	0.0219 (−55.2%)	0.0880 (−10.6%)	0.0797 (56.2%)	0.0426 (−57.6%)
0.044 (−30%)	0.0280 (−42.6%)	0.0918 (−6.7%)	0.0758 (48.6%)	0.0532 (−47.0%)
0.051 (−20%)	0.0355 (−27.2%)	0.0950 (−3.3%)	0.0681 (33.6%)	0.0678 (−32.4%)
0.054 (−15%)	0.0388 (−20.4%)	0.0962 (−2.2%)	0.0642 (25.8%)	0.0735 (−25.3%)
0.057 (−10%)	0.0421 (−13.7%)	0.0971 (−1.3%)	0.0599 (17.5%)	0.0827 (−17.6%)
0.060 (−5%)	0.0450 (−6.8%)	0.0980 (−0.6%)	0.0555 (8.8%)	0.0912 (−9.1%)
0.063 (0%)	0.0488 (0.0%)	0.0984 (0.0%)	0.0510 (0.0%)	0.1000 (0.0%)
0.066 (5%)	0.0522 (6.8%)	0.0988 (0.3%)	0.0464 (−8.8%)	0.1100 (9.9%)
0.069 (10%)	0.0555 (13.6%)	0.0985 (0.6%)	0.0421 (−17.5%)	0.1210 (20.6%)
0.072 (15%)	0.0587 (20.3%)	0.0992 (0.8%)	0.0377 (−26.0%)	0.1328 (32.2%)
0.076 (20%)	0.0630 (29.1%)	0.0992 (0.7%)	0.0322 (−36.7%)	0.1497 (49.1%)
0.082 (30%)	0.0693 (42.0%)	0.0988 (0.35%)	0.0248 (−51.3%)	0.1784 (77.6%)
0.088 (40%)	0.0753 (54.4%)	0.0979 (0.5%)	0.0186 (−63.6%)	0.2110 (110.2%)
0.094 (49%)	0.0810 (66.0%)	0.0965 (1.0%)	0.0134 (−73.7%)	0.2479 (146.8%)

**Table 3:** Effect of $\sigma_{p}$ on producer and consumer risks in AR and BR designs, with $\tau_{1}=0.05$, $\tau_{2}=0.10$, $p_{\tau_{1}}=0.0319$, $p_{\tau_{2}}=0.0942$, $\mu_{p}=0.063$, and $\sigma_{p}=0.054$ under 50% censoring.

$\sigma_{p}$	AR Design		BR Design	
	APR	ACR	BPR	BCR
0.036 (−33%)	0.0783 (60.4%)	0.1316 (33.7%)	0.0417 (−18.3%)	0.0967 (−3.6%)
0.041 (−25%)	0.0684 (40.1%)	0.1208 (22.7%)	0.0465 (−8.9%)	0.1012 (0.7%)
0.044 (−18%)	0.0631 (29.3%)	0.1150 (16.8%)	0.0484 (−5.1%)	0.1023 (1.9%)
0.048 (−12%)	0.0568 (16.5%)	0.1078 (9.5%)	0.0500 (−1.9%)	0.0827 (2.0%)
0.051 (−6%)	0.0527 (7.8%)	0.1029 (4.6%)	0.0507 (−0.5%)	0.1020 (1.3%)
0.054 (0%)	0.0488 (0.0%)	0.0984 (0.0%)	0.0510 (0.0%)	0.1000 (0.0%)
0.057 (5%)	0.0453 (−7.1%)	0.0942 (−4.3%)	0.0510 (0.0%)	0.0987 (−1.6%)
0.060 (11%)	0.0421 (−13.6%)	0.0902 (−8.3%)	0.0508 (−0.4%)	0.0967 (−3.7%)
0.063 (15%)	0.0392 (−19.6%)	0.0866 (−12.0%)	0.0503 (−1.3%)	0.0944 (−5.9%)
0.065 (20%)	0.0374 (−23.3%)	0.0843 (−14.3%)	0.0500 (−1.9%)	0.0928 (−7.6%)
0.068 (25%)	0.0349 (−28.4%)	0.0810 (−17.6%)	0.0494 (−3.2%)	0.0902 (−10.2%)

**Table 4:** Effect of $\mu_{p}$ on producer and consumer risks in AR and BR designs, with $\tau_{1}=0.05$, $\tau_{2}=0.10$, $p_{\tau_{1}}=0.0319$, $p_{\tau_{2}}=0.0942$, $\mu_{p}=0.063$, and $\sigma_{p}=0.054$ under 10% censoring.

$\mu_{p}$	AR Design		BR Design	
	APR	ACR	BPR	BCR
0.032 (−49%)	0.0170 (−66.7%)	0.0855 (−15.6%)	0.0890 (59.1%)	0.0366 (−66.2%)
0.038 (−40%)	0.0213 (−55.1%)	0.0910 (−10.5%)	0.0882 (57.7%)	0.0466 (−56.9%)
0.044 (−30%)	0.0293 (−42.5%)	0.0946 (−6.6%)	0.0837 (49.7%)	0.0580 (−46.5%)
0.051 (−20%)	0.0371 (−27.1%)	0.0980 (−3.3%)	0.0751 (34.2%)	0.0737 (−32.0%)
0.054 (−15%)	0.0405 (−20.4%)	0.0991 (−2.2%)	0.0706 (26.3%)	0.0814 (−24.9%)
0.057 (−10%)	0.0440 (−13.6%)	0.1000 (−1.3%)	0.0659 (17.8%)	0.0897 (−17.3%)
0.060 (−5%)	0.0475 (−6.8%)	0.1000 (−0.6%)	0.0609 (9.0%)	0.0987 (−9.0%)
0.063 (0%)	0.0509 (0.0%)	0.1014 (0.0%)	0.0559 (0.0%)	0.1084 (0.0%)
0.066 (5%)	0.0544 (6.8%)	0.1018 (0.4%)	0.0510 (−8.7%)	0.1189 (9.7%)
0.069 (10%)	0.0578 (13.5%)	0.1021 (0.7%)	0.0461 (−17.6%)	0.1305 (20.2%)
0.072 (15%)	0.0612 (20.1%)	0.1022 (0.9%)	0.0413 (−26.1%)	0.1426 (31.5%)
0.076 (20%)	0.0656 (28.8%)	0.1022 (0.9%)	0.0353 (−36.8%)	0.1605 (48.0%)
0.082 (30%)	0.0720 (42.0%)	0.1020 (0.4%)	0.0271 (−51.4%)	0.1905 (75.7%)
0.088 (40%)	0.0783 (53.7%)	0.1009 (0.5%)	0.0202 (−63.8%)	0.2246 (107.1%)
0.094 (49%)	0.0841 (66.0%)	0.1015 (1.0%)	0.0146 (−73.8%)	0.2628 (142.4%)

**Table 5:** Effect of $\sigma_{p}$ on producer and consumer risks in AR and BR designs, with $\tau_{1}=0.05$, $\tau_{2}=0.10$, $p_{\tau_{1}}=0.0319$, $p_{\tau_{2}}=0.0942$, $\mu_{p}=0.063$, and $\sigma_{p}=0.054$ under 10% censoring.

$\sigma_{p}$	AR Design		BR Design	
	APR	ACR	BPR	BCR
0.036 (−33%)	0.0813 (59.6%)	0.1351 (33.3%)	0.0452 (−19.1%)	0.1030 (−5.2%)
0.041 (−25%)	0.0711 (39.6%)	0.1241 (22.5%)	0.0506 (−9.4%)	0.1080 (−0.3%)
0.044 (−18%)	0.0657 (29.0%)	0.1182 (16.6%)	0.0528 (−6.0%)	0.1095 (1.1%)
0.048 (−12%)	0.0592 (16.3%)	0.1109 (9.4%)	0.0540 (−2.2%)	0.1102 (1.6%)
0.051 (−6%)	0.0549 (7.7%)	0.1060 (4.6%)	0.0507(−0.5%)	0.1020 (1.3%)
0.054 (0%)	0.0509 (0.0%)	0.1014 (0.0%)	0.0559 (0.0%)	0.1084 (0.0%)
0.057 (5%)	0.0473 (−7.1%)	0.0970 (−4.2%)	0.0561 (0.3%)	0.1068 (−1.5%)
0.060 (11%)	0.0440 (−13.6%)	0.0930 (−8.2%)	0.0559 (0.0%)	0.1049 (−3.2%)
0.063 (15%)	0.0409 (−19.5%)	0.0893 (−11.8%)	0.0555 (−0.7%)	0.1026 (−5.3%)
0.065 (20%)	0.0391 (−23.2%)	0.0870 (−14.2%)	0.0551 (−1.4%)	0.1010 (−6.8%)
0.068 (25%)	0.0365 (−28.4%)	0.0836 (−17.5%)	0.0545 (−2.6%)	0.0984 (−9.2%)

**Table 6:** Effect of $\mu_{p}$ on producer and consumer risks in AR and BR designs, with $\tau_{1}=0.05$, $\tau_{2}=0.10$, $p_{\tau_{1}}=0.0319$, $p_{\tau_{2}}=0.0942$, $\mu_{p}=0.063$, and $\sigma_{p}=0.054$ under 10% censoring.

$\mu_{p}$	AR Design		BR Design	
	APR	ACR	BPR	BCR
0.032 (−49%)	0.0161 (−66.9%)	0.0827 (−15.7%)	0.0789 (57.1%)	0.0333 (−66.8%)
0.038 (−40%)	0.0218 (−55.2%)	0.0876 (−10.6%)	0.0784 (56.0%)	0.0425 (−57.6%)
0.044 (−30%)	0.0279 (−42.7%)	0.0915 (−6.7%)	0.0746 (48.5%)	0.0530 (−47.0%)
0.051 (−20%)	0.0371 (−27.3%)	0.0948 (−3.3%)	0.0671 (33.5%)	0.0676 (−32.5%)
0.054 (−15%)	0.0387 (−20.5%)	0.0959 (−2.2%)	0.0632 (25.7%)	0.0748 (−25.3%)
0.057 (−10%)	0.0420 (−13.7%)	0.0968 (−1.3%)	0.0590 (17.4%)	0.0825 (−17.6%)
0.060 (−5%)	0.0453 (−6.9%)	0.0975 (−0.6%)	0.0547 (8.8%)	0.0910 (−9.1%)
0.063 (0%)	0.0487 (0.0%)	0.0981 (0.0%)	0.0502 (0.0%)	0.1001 (0.0%)
0.066 (5%)	0.0520 (6.8%)	0.0984 (0.4%)	0.0458 (−8.8%)	0.1100 (9.9%)
0.069 (10%)	0.0553 (13.6%)	0.0987 (0.7%)	0.0414 (−17.5%)	0.1208 (20.6%)
0.072 (15%)	0.0586 (20.3%)	0.0988 (0.8%)	0.0372 (−26.0%)	0.1324 (32.2%)
0.076 (20%)	0.0629 (29.1%)	0.0988 (0.8%)	0.0318 (−36.7%)	0.1493 (49.1%)
0.082 (30%)	0.0691 (42.0%)	0.0984 (0.4%)	0.0245 (−51.2%)	0.1778 (77.6%)
0.088 (40%)	0.0751 (54.3%)	0.0975 (−0.5%)	0.0183 (−63.6%)	0.2104 (110.2%)
0.094 (49%)	0.0808 (66.0%)	0.0962 (−1.8%)	0.0132 (−73.7%)	0.2472 (146.9%)

**Table 7:** Effect of $\sigma_{p}$ on producer and consumer risks in AR and BR designs, with $\tau_{1}=0.05$, $\tau_{2}=0.10$, $p_{\tau_{1}}=0.0319$, $p_{\tau_{2}}=0.0942$, $\mu_{p}=0.063$, and $\sigma_{p}=0.054$ under 90% censoring.

$\sigma_{p}$	AR Design		BR Design	
	APR	ACR	BPR	BCR
0.036 (−33%)	0.0780 (60.3%)	0.1312 (33.8%)	0.0411 (−18.1%)	0.0965 (−3.6%)
0.041 (−25%)	0.0682 (40.0%)	0.1204 (22.7%)	0.0459 (−8.7%)	0.1009 (0.8%)
0.044 (−18%)	0.0630 (29.3%)	0.1146 (16.8%)	0.0477 (−5.0%)	0.1020 (1.9%)
0.048 (−12%)	0.0567 (16.4%)	0.1075 (9.5%)	0.0493 (−1.9%)	0.1021 (2.0%)
0.051 (−6%)	0.0525 (7.8%)	0.1026 (4.6%)	0.0500 (−0.6%)	0.1013 (1.2%)
0.054 (0%)	0.0487 (0.0%)	0.0981 (0.0%)	0.0502 (0.0%)	0.1001 (0.0%)
0.057 (5%)	0.0452 (−7.2%)	0.0939 (−4.3%)	0.0502 (−0.0%)	0.0984 (−1.7%)
0.060 (11%)	0.0420 (−13.7%)	0.0899 (−8.3%)	0.0499 (0.4%)	0.0964 (−3.7%)
0.063 (15%)	0.0391 (−19.6%)	0.0863 (−11.9%)	0.0496 (−1.3%)	0.0941 (−6.0%)
0.065 (20%)	0.0373 (−23.3%)	0.0840 (−14.3%)	0.0492 (−2.0%)	0.0924 (−7.6%)
0.068 (25%)	0.0348 (−28.5%)	0.0808 (−17.6%)	0.0486 (−3.3%)	0.0899 (−10.2%)

## Data Availability

The data presented in this study are openly available in [26].
